# Mechanistic Understanding of the Olfactory Neuroepithelium Involvement Leading to Short-Term Anosmia in COVID-19 Using the Adverse Outcome Pathway Framework

**DOI:** 10.3390/cells11193027

**Published:** 2022-09-27

**Authors:** Muhammad Ali Shahbaz, Francesca De Bernardi, Arto Alatalo, Magdalini Sachana, Laure-Alix Clerbaux, Amalia Muñoz, Surat Parvatam, Brigitte Landesmann, Katja M. Kanninen, Sandra Coecke

**Affiliations:** 1 A.I. Virtanen Institute for Molecular Sciences, University of Eastern Finland, 70211 Kuopio, Finland; 2 Division of Otorhinolaryngology, Department of Biotechnologies and Life Sciences, University of Insubria, Ospedale di Circolo e Fondazione Macchi, 21100 Varese, Italy; 3 Environment Health and Safety Division, Environment Directorate, Organisation for Economic Cooperation and Development (OECD), 75775 Paris, France; 4 European Commission, Joint Research Centre (JRC), 21027 Ispra, Italy; 5 European Commission, Joint Research Centre (JRC), 2440 Geel, Belgium; 6 Centre for Predictive Human Model Systems, Atal Incubation Centre-Centre for Cellular and Molecular Biology (AIC-CCMB), Habsiguda, Hyderabad 500039, India

**Keywords:** SARS-CoV-2 infection, COVID-19, anosmia, olfactory neuroepithelium, AOP

## Abstract

Loss of the sense of smell (anosmia) has been included as a COVID-19 symptom by the World Health Organization. The majority of patients recover the sense of smell within a few weeks postinfection (short-term anosmia), while others report persistent anosmia. Several studies have investigated the mechanisms leading to anosmia in COVID-19; however, the evidence is scattered, and the mechanisms remain poorly understood. Based on a comprehensive review of the literature, we aim here to evaluate the current knowledge and uncertainties regarding the mechanisms leading to short-term anosmia following SARS-CoV-2 infection. We applied an adverse outcome pathway (AOP) framework, well established in toxicology, to propose a sequence of measurable key events (KEs) leading to short-term anosmia in COVID-19. Those KEs are (1) SARS-CoV-2 Spike proteins binding to ACE-2 expressed by the sustentacular (SUS) cells in the olfactory epithelium (OE); (2) viral entry into SUS cells; (3) viral replication in the SUS cells; (4) SUS cell death; (5) damage to the olfactory sensory neurons and the olfactory epithelium (OE). This AOP-aligned approach allows for the identification of gaps where more research should be conducted and where therapeutic intervention could act. Finally, this AOP gives a frame to explain several disease features and can be linked to specific factors that lead to interindividual differences in response to SARS-CoV-2 infection.

## 1. Introduction

The World Health Organization (WHO) declared the Coronavirus disease 19 (COVID-19) outbreak a pandemic in March 2020. The sudden and storming onset of severe acute respiratory syndrome coronavirus 2 (SARS-CoV-2) infection is primarily associated with the respiratory syndrome. However, a wide range of neurological symptoms, such as a loss of the sense of smell (anosmia) or taste (ageusia), stroke, paralysis, cranial nerve deficits, encephalopathy, meningitis, delirium, and seizures have also been reported accompanying the disease and affecting its course.

Olfactory dysfunction (OD), including anosmia, has been included as a symptom of COVID-19 by the WHO. However, the underlying mechanisms leading to anosmia in patients with COVID-19 remain poorly understood; therefore, therapeutic interventions for anosmia remain inadequate. Based on a comprehensive review of the literature, we aim here to determine the causal sequence of the biological steps leading to anosmia following SARS-CoV-2 infection: from the initial interaction of the virus at the molecular level with the olfactory neuroepithelium up to the adverse outcome of anosmia. We applied the AOP framework to describe this sequence of events. The AOP framework is well-established in the toxicological field and is used for chemical risk assessment and regulatory purposes [1,2]. However, the AOPs are also foreseen to be of great value for biomedical research [3]. Currently, diseases are mainly classified based on symptoms. A mechanistically based classification of the disease, presenting the same symptoms, could help to provide more efficient therapeutic interventions [4].

An AOP is intentionally simplifying complex biology, proposing a biological pathway from an initial event up to an adverse outcome with a set of methods to assess the level of confidence in the proposed mechanisms. AOPs do not describe every detail of the pathological process in question but rather focus on the critical steps, the key events (KE), which are measurable, and have potential predictive value for the respective AOP [5,6]. Applying the AOP framework to depict the pathological mechanisms leading to OD in COVID-19 will help to identify the biological KEs along the path to adversity, where therapeutic intervention could act. AOPs also allow for identifying knowledge gaps to strengthen understanding of the COVID-19-associated OD. Finally, AOPs give a structured frame to explain several of the disease features and can be linked to specific modulating factors that lead to the interindividual differences in response to SARS-CoV-2 infection.

### 1.1. Anosmia and COVID-19

Anosmia is a complete loss of olfactory functions resulting in a complete loss of ability to smell odors, while partial anosmia is a quantitatively reduced ability to smell a specific odor despite the preserved ability to smell most other odors. Chemosensory dysfunctions also include parosmias (changed perceptions of odors) and hypogeusia (reduced ability to taste things) [7]. However, it is postulated that in cases with COVID-19 infection, ageusia might be a secondary consequence of anosmia [8]. According to a systematic review and meta-analysis published in January 2021, of 32,142 COVID-19 patients from 107 studies, 38.2% reported a prevalence of anosmia (95% CI: 36.5%, 47.2) [9]. However, the paper indicated that prevalence varies across different studies, with a significant difference in the prevalence between western countries and southeast Asian countries. The variation in prevalence was attributed to both host and viral factors. Increasing evidence over the last two years suggests that host genetics, age, ethnicity, and geographical location might impact the occurrence of anosmia in COVID-19 [10,11,12]. Meanwhile, continuous viral evolution in the Spike protein RBD and the emergence of new variants influence the anosmia incidence rate [13].

In the early part of the pandemic, anosmia was not considered to be a common complication of COVID-19. However, the prevalence of anosmia increased in the SARS-CoV-2-infected individuals with its spread across the globe and the emergence of the D614G variant (mutation in the original Wuhan strain D614) [13,14]. Since the start of the pandemic, SARS-CoV-2 has evolved through the appearance of several variants. According to the WHO, variants of concern are Alpha (B.1.1.7), Beta (B.1.351), Gamma (P.1), Delta (B.1.617.2 and AY lineages), and Omicron (originally B.1.1.529, then reclassified into BA lineages) [15]. Several studies have investigated the effects of the developing variants on the prevalence of anosmia in COVID-19 patients [16,17,18,19,20,21]. A recently published review comparing data published on the contribution of genetic variants to the incidence of anosmia concluded that the Omicron variant causes much less OD than the other variants [22]. Furthermore, it is also suggested that minor spike mutations in Alpha, Beta, and Delta variants slightly altered the SARS-CoV-2 viral ability to induce chemosensory deficits [22]. Modification in the SARS-CoV-2 Spike protein potentially changes cell tropism and interaction with proteins that promote virus uptake [23].

Anosmia has been classified into short-term anosmia and persistent or long-term anosmia in patients with COVID-19, and the course is related to modulating factors: age, sex, and genetic factors [24]. In both cases, the onset is often sudden, regardless of other features. The majority of patients have a complete or extensive improvement in their sense of smell within 2–3 weeks after the first symptoms [25,26]. However, in about 10–20% of the cases, loss of smell persists longer. Clinical data from COVID-19 recovered cases with persistent anosmia showed recovery of the sense of smell within a few months after the onset of infection [26]. However, in some individuals, OD persists even one year postinfection [27]. In this review, we have particularly focused on KEs that follow COVID-19 infection and lead to short-term anosmia.

### 1.2. The Olfactory Mucosa

#### 1.2.1. Anatomy

**Figure 1:**
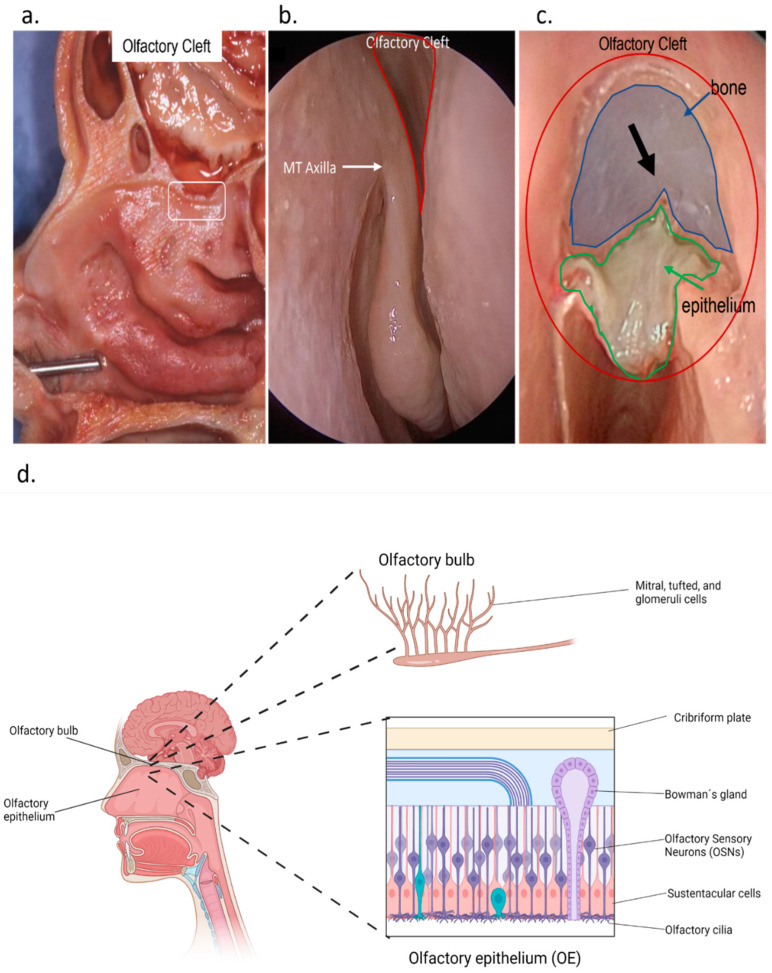
Human Olfactory Neuroepithelium; (**a**) Sagittal section of an anatomical specimen showing in the cranial portion of the nasal cavity (in the box) the area where the OE is positioned. The epithelium rests on the cribriform plate of the ethmoidal bone, which is crossed by the olfactory fibers which connect with the olfactory bulb; (**b**) endonasal view of the olfactory cleft (circled in red), the area where the OE is located (MT axilla—axilla of the middle turbinate); (**c**) endonasal vision after lifting the epithelium (circled in green) from the bone (circled in blue) to highlight the first olfactory fiber (black arrow); (**d**) schematic representation of the location and composition of the OE. (**a**–**c**) are taken by the Head and Neck Surgery & Forensic Dissection Research Center (HNS & FDRC), Department of Biotechnology and Life Sciences, University of Insubria, Varese, Italy.

The olfactory mucosa lies within the olfactory cleft. The olfactory cleft is an anatomic region demarcated by the cribriform plate superiorly, superior turbinate laterally, and superior septum medially. Patients with COVID-19-related olfactory disorders have larger olfactory cleft widths and volumes than those without OD [28]. In the olfactory cleft, there is the olfactory epithelium (OE) (Figure 1). The OE is situated on the rooftop of the nasal cavity, directly exposed to the environment. It harbors a heterogeneous population of cells responsible for olfactory sensations. There are at least five distinct cell types present in the OE: olfactory sensory neurons (OSNs), sustentacular (SUS) cells, microvillar cells, duct cells of the olfactory (Bowman’s) glands, and basal cells [29]. Deep in the OE and separated by the basement membrane, the lamina propria contains a dense vascular network as well as Bowman glands, connective tissue, and olfactory ensheathing cells (a specialized form of glia). Taken together, the lamina propria and OE constitute the olfactory mucosa [30,31]. Lying above the olfactory mucosa is the olfactory bulb, the most important part of the primary olfactory brain network. Axons from the olfactory sensory neurons enter the ipsilateral olfactory bulb and synapse with second-order neurons, called mitral and tufted cells, within specialized regions called glomeruli. The axons of the second-order olfactory neurons subsequently project to diverse olfactory areas of the central nervous system (CNS) [32]. Collectively, these structures make up the primary olfactory network.

#### 1.2.2. Olfactory Physiology

Initially, odorants enter the superior aspect of the nasal cavity, where the olfactory mucosa plays a key role in the perception of smell through olfactory sensory neurons, as they possess sensory cilia with odorant receptors (OR). From each dendritic knob of an OSN, 10–30 cilia protrude out into the mucus layer. The odorants bind to ORs on the cilia. ORs are G-protein-coupled receptors that are responsible for the generation of action potentials upon binding to odorants. This action potential propagates through axons of all OR-specific OSNs to the glomerulus, where the axons synapse with mitral and tufted cells in the olfactory bulb. Initial processing of the signal happens in the olfactory bulb [33,34]. Once the information is processed, the axons of the second-order olfactory neurons (mitral and tufted cells) subsequently send information to other areas of the CNS, which are involved in further processing, modulation, and interpretation of smell [32].

## 2. Underlying Biological Mechanisms Leading to Short-Term Anosmia in COVID-19

ODs can be broadly categorized into conductive or sensorineural olfactory loss [35]. Conductive loss occurs due to impaired nasal airflow and is reversible when the obstruction clears. Sensorineural loss implies dysfunction of the OE and can be permanent or have a longer time course to functional recovery of a sense of smell [36].

Initially, some studies on COVID-19 patients suggested anosmia to be due to conductive loss by localized inflammation in the OE [37,38,39]. Moreover, the transient onset of OD after infection with SARS-CoV-2 and recovery of symptoms within 8 days of the infection could possibly be supportive of a conductive mechanism. However, no significant association between sinonasal symptoms and anosmia had been identified, suggesting that the pathogenesis of COVID-19-related anosmia might differ from obstructive OD seen in other viral upper respiratory tract infections [40].

**Figure 2:**
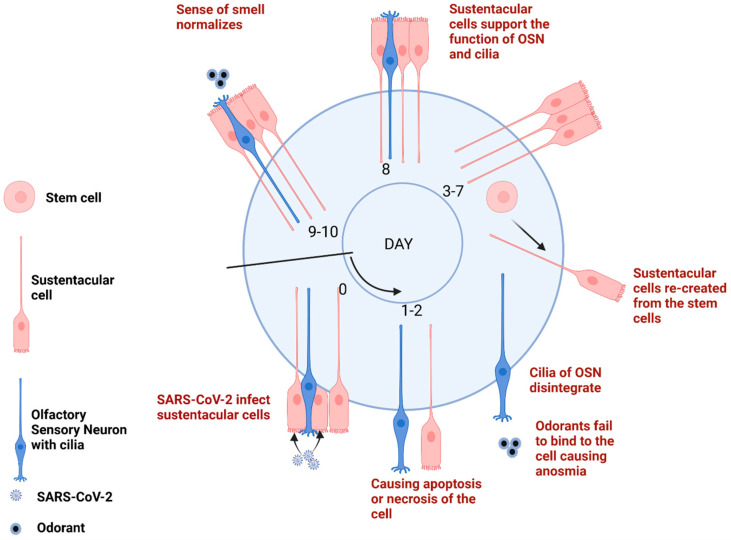
Temporal representation of the pathological mechanisms leading to short-term anosmia following SARS-CoV-2 infection. Created with Biorender.com (access on 1 August 2022).

Anosmia caused by a sensorineural loss in COVID-19 might be induced following the binding of SARS-CoV-2 to cells and damage to different cell types of the OE. SUS cells seem to be the main target of SARS-CoV-2 in the olfactory system [41]. Over the last two years, several studies have been published to investigate the different plausibility associated with SARS-CoV-2 effects on the OE. Here, we have developed an AOP for short-term anosmia based on the current knowledge and understanding of OD in COVID-19. The aim of developing an AOP for short-term anosmia is to better understand the mechanisms underlying the anosmia’s adverse outcome. Mapping of the interaction of SARS-CoV-2 at the molecular level and the subsequent cellular and tissue events give a structured framework to explain disease features. Temporal representation of the sequence of events that follow in short-term anosmia caused by COVID-19 infection is demonstrated in Figure 2.

## 3. OE Damage Underlying Short-Term Anosmia

### 3.1. S Protein Binding to ACE2 in SUS Cells Leads to Increased Viral Entry

#### 3.1.1. Biological Plausibility

Infection with SARS-CoV-2 initiates with the binding of the viral Spike (S) protein to the angiotensin-converting enzyme 2 (ACE2) plasma membrane surface receptor (KE1739). Upon binding to ACE2, the viral S proteins are activated through proteolytic cleavage to allow membrane fusion—a key step in viral entry. Many proteases that are responsible for the proteolytic cleavage of the S protein of coronaviruses have been identified, including transmembrane serine protease 2 (TMPRSS2) [42,43,44,45], cathepsin L (CTSL [46,47], and furin [48].

#### 3.1.2. Empirical Evidence

The OE consists of a heterogeneous cell population, including the olfactory sensory neurons (OSNs), basal, SUS cells, and microvillar and glandular cells. One or several of these cell types could serve as a potential point of viral entry into the OE and thus determine the underlying chain of events leading to anosmia.

In the OE, ACE2 has been reported to be present in humans and several animals and in ex vivo and in vitro models. In summary, the literature to date has shown ACE-2 and TMPRSS2 to be expressed in SUS cells (Table 1), Bowman’s gland, and microvillar cells but not in the OSNs [49,50,51,52,53]. Importantly, most studies have reported that ACE-2 is particularly highly expressed in the SUS cells of the OE [51,52]. Furthermore, TMPRSS2, which is a well-established host factor in facilitating SARS-CoV-2 entry upon binding to ACE-2, has also been reported to be expressed in the SUS cells [54,55]. Fodoulian et al. showed coexpression of ACE-2 and TMPRSS2 in a subset of the SUS cell population of the human OE [52].

In addition to the abovementioned evidence for ACE-2 facilitated viral entry, some studies have suggested the Neuropilin-1 receptor (NRP-1) as an alternative receptor for SARS-CoV-2 entry (KE1738) [56]. NRP-1 is expressed in all cell types of the OE, including the OSNs, and its expression level is higher than that of ACE-2 in the OE and olfactory bulb [57,58]. NRP-1 is thus implicated in SARS-CoV-2 entry in OSN. In addition to its role in viral entry, Mayi et al. (2021) described that the NRP-1 malfunction is implicated in Kallmann syndrome, a congenital disease characterized by hypogonadism and anosmia [59]. Together, the involvement of NRP-1 in SARS-CoV-2 entry and its association with anosmia support the presence of an alternative route of SARS-CoV-2 entry and CNS manifestation. However, a recent review by Jakson et al. reported higher expression of NRP-1 in goblet cells, a cell population not susceptible to SARS-CoV-2, than in ciliated cells of the olfactory epithelium that are considered a major target for SARS-CoV-2. [60]. Further studies are thus required to confirm the role of NRP1 in SARS-CoV-2-induced anosmia and neurological manifestations.

#### 3.1.3. Modulating Factors

To date, one study carried out in mice has shown that the levels of ACE2 and TMPRSS2 proteins are increased in the OE upon aging. This may explain why older animals (and humans) are more susceptible to SARS-CoV-2 infection [54]. According to Baker SA et al. (2021), human lung ACE2 expression is strongly correlated with age in patients that require mechanical ventilation during SARS-CoV-2 infection. The patients that did not require mechanical ventilation did not show changes in ACE2 expression with age [61]. Similarly, a meta-analysis using single-cell RNA-sequencing of human nasal, airway, and lung parenchyma tissues revealed cell type-specific associations of age, sex, and smoking with expression levels of ACE2, TMPRSS2, and CTSL [62]

#### 3.1.4. Overall Assessment

Based on the existing evidence and the context of this AOP, there is strong evidence that the SARS-CoV-2 S protein binds to ACE2 in SUS cells and enters the cells by priming with TMPRSS2 and other proteases. Sensory neurons of the olfactory system do not express ACE2. NRP-1 is also indicated as a potential alternative for viral entry in the OSNs and olfactory bulb; however, further evidence is needed for confirmation.

**Table 1:** Evidence for expression of ACE2 receptor in the OE and specifically SUS cells. Hu—Human, Mo—Mouse, Ha—Hamster, S1—in vitro, S2—in vivo, S3—ex vivo, S4—in silico. KE1739 and KE1738 refer to (https://aopwiki.org/ (Accessed on 20 September 2022)).

KE1739 and KE1738								
Observations	Model Organism			Type of Study				Ref.
	Hu	Mo	Ha	S1	S2	S3	S4	
**ACE2 is expressed in the SUS cells of the OE to elucidate the symptoms of SARS-CoV-2-induced anosmia via the olfactory pathway**	☒	☐	☐	☒	☐	☐	☐	[49]
**Subsets of human OE SUS cells (basal and Bowman’s gland cells) express the SARS-CoV-2 receptor ACE2**	☒	☐	☐	☒	☐	☐	☐	[53]
**ACE2 expression confirmed** **in OE in** **humans**	☒	☐	☐	☒	☐	☐	☐	[63]
**The upper airway is the initial site of SARS-CoV-2 infection based on data on ACE2 expression in the SUS cells**	☒	☐	☐	☒	☐	☐	☐	[51]
**ACE2 protein is highly expressed in a subset of SUS cells in human olfactory tissues**	☒	☐	☐	☒	☐	☐	☐	[52]
***Meta-analysis indicates that ACE2*** **is enriched within OE subsets**	☒	☐	☐	☐	☐	☐	☒	[64]
**Single-cell RNA-sequencing uncovers putative targets of SARS-CoV-2 among tissue-resident cell subsets.**	☐	☒	☐	☐	☐	☐	☒	[64]
**Subsets of mouse OE SUS cells,** **horizontal basal cells (HBCs), and Bowman’s gland cells express the ACE2 receptor**	☐	☒	☐	☒	☐	☐	☐	[53]
**ACE2 is expressed in the** **supporting cells and Bowman’s gland of the mouse Olfactory Mucosa (OM)** **, except for the cilia in the respiratory epithelium of mice**	☐	☒	☐	☒	☐	☐	☐	[63]
**ACE2 protein is highly expressed in a subset of SUS cells in human and mouse olfactory tissues**	☐	☒	☐	☒	☐	☐	☐	[52]
**SUS cells are identified as the main target cells of SARS-CoV-2 in the OE, which highly express ACE2**	☒	☐	☐	☐	☐	☒	☐	[41]

### 3.2. Viral Entry in SUS Cells Leads to Increased SARS-CoV-2 Production

#### 3.2.1. Biological Plausibility

As described above, SUS cells express high levels of molecules associated with SARS-CoV-2 entry. Following the entry of SARS-CoV-2 into the SUS cells (KE1738), the virus replicates in the cells.

#### 3.2.2. Empirical Evidence

Over the last two years, several studies have demonstrated infection of SARS-CoV-2 in the OE, predominantly in the SUS cells. A study carried out on brush samples of the olfactory mucosa of SARS-CoV-2-infected anosmia patients showed active replication of SARS-CoV-2. However, this study did not characterize viral replication in the OE cell subtypes [65]. Khan et al. (2021) reported SARS-CoV-2 load in non-neural OE, mainly in the SUS cells surrounding the olfactory sensory neurons, using special transcriptomics and immunohistochemistry on postmortem tissues of human olfactory mucosa and OB [41]. The study also reported that 30% of the individuals had active replication in the SUS cells of the postmortem tissue autopsies; however, none of the investigated biopsies of the olfactory bulb showed signs of the virus. Furthermore, similar findings have been reported in golden Syrian hamsters, where massive damage of the OE was observed and associated with infection of a large proportion of SUS cells [50]. Similarly, in a mouse model, the virus was shown to be present in the OE on day 2, already decreasing by day 4, primarily with an infection of the supporting SUS cells but not the olfactory neurons themselves [66].

#### 3.2.3. Modulating Factors

A very recent study showed in vitro (human explants of OE cells) and in vivo (hamsters) that the wild-type and Delta variant of SARS-CoV-2 have broader cellular invasion capability in olfactory submucosa as it infects a proportion of olfactory neurons in addition to the primary target sustentacular cells, whereas the Omicron variant may infect the SUS cells but heavily retained in the sinonasal epithelium [67]. Similarly, Omicron caused milder pathological changes in the upper and lower respiratory tract compared with other SARS-CoV-2 variants in Syrian golden hamsters with intranasal inoculation of the different variants [68]. This could potentially explain differences in percentages of anosmia cases caused by SARS-CoV-2 infection caused by different variants. However, further investigations are needed for a clear understanding of the effects of individual SARS-CoV-2 variants on the olfactory system.

#### 3.2.4. Overall Assessment

In the OE, it has been proven that in human pathology, mouse, and other in vivo models SARS-CoV-2 mainly replicates in SUS cells. However, to date, the low numbers of published studies mean limited knowledge of the molecular events of viral replication in SUS cells of the OE.

### 3.3. SARS-CoV-2 Production Leads to SUS Cells Decrease

#### 3.3.1. Biological Plausibility

SUS cells provide functional and structural support to the OE, including the olfactory neurons. Anatomically, the SUS cells are in close proximity to the OSNs, and it has been proposed that they play an important role in the perception of smells or olfaction mechanism by peripheral processing of odorants [10,50,69]. In addition, these cells express enzymes of the cytochrome P450 family, which are involved in the detoxification of volatile chemicals that are potentially damaging to the OSNs [70]. Moreover, these cells perform endocytosis of the odorant-binding protein complexes after signal transduction at the neuronal cilia. Clearance of the odorant receptors increases sensitivity for further odorant reception [70,71]. A further function of the SUS cells is their involvement in the innate immune response to a foreign assault by the generation of inflammatory cytokines. These cells have been reported to secrete cytokines and chemokines such as TNF, IL-1β, IL-1α, and CXCL2 in patients with chronic rhinosinusitis and COVID-19 [50,72,73]. In addition to the mentioned roles, SUS cells also manage glucose transport and local salt and water balance to OSNs [73]. Given the multitude of important roles of the SUS cells, the loss of these cells can impair the function of the olfactory system. Therefore, robust replication leading to the death of SUS cells during SARS-CoV-2 infection may provide a plausible explanation for the reason behind the loss of smell (notherKE1870) and strong support for the essentiality of this KE in the pathway.

#### 3.3.2. Empirical Evidence

SARS-CoV-2 infection of the OE results in the downregulation of specific cell markers for SUS cells in Syrian hamsters on day 2, and this reduction peaks on day 4 of infection. Furthermore, single-cell RNA-sequencing of the SARS-CoV-2-infected OE demonstrates a depletion of infected cells [74]. In addition, intranasal inoculation of SARS-CoV-2 in a transgenic mouse model expressing human ACE2 causes massive cell death and apoptosis in the OE and lamina propria of the olfactory system, and apoptosis of the SUS cells [50,63].

#### 3.3.3. Modulating Factors

UGT2A1 and UGT2A2 genes are expressed in the OE that play a critical role in the physiology of olfaction. They serve in odorant function as odorant metabolizing enzymes, facilitating the elimination of exogenous and endogenous compounds. A recent study identified a strong association at a locus containing the UGT2A1 and UGT2A2 genes with COVID-19-related anosmia [75]. This study also identified variants of UGT2A1 and UGT2A2 genes in SARS-CoV-2-infected patients with loss of sense of smell. Furthermore, studies showed SUS cells heavily express UGT2A1 [41,76]. This may explain the genetic basis of the fact that not every individual infected with SARS-CoV-2 experiences a loss of olfaction. Evidence shown in KER3 suggests that SARS-CoV-2 impedes olfactory sensation by infecting and compromising the essential functions of olfactory support cells, which may be modulated by UGT2A1/UGT2A2.

#### 3.3.4. Overall Assessment

Emerging evidence suggests that SUS cells are the primary target of the SARS-CoV-2 virus and that infection leads to SUS cell death.

### 3.4. SUS Cells Decrease Leads to Reduction in the Olfactory Sensory Neurons

#### 3.4.1. Biological Plausibility

As described, SUS cells are closely associated, both metabolically and functionally, with OSNs and with odorant signal transduction. The death of these supporting cells can lead to cascading changes in the structure and function of the OE [51,77,78]. There are several possible scenarios that can develop in response to the damage to the SUS cell population. One possibility is that the loss of SUS cells caused by SARS-CoV-2 infection can inhibit the perception of odorants in adjacent OSNs [54]. Furthermore, SUS cell death can also lead to the loss of cilia of olfactory sensory neurons and deficient signal transduction [50]. Another possibility is that, since the supporting cells of the OE regulate glucose transport to the OSN by balancing local water and ion balance, damage to them can influence signaling from the olfactory neurons to the brain [72]. Finally, the secretion of proinflammatory cytokines by SUS cells infected with SARS-CoV-2 may impede collateral damage to olfactory sensory neurons. 

#### 3.4.2. Empirical Evidence

Early studies after the onset of the SARS-CoV-2 pandemic provided the initial evidence that the OSNs do not express SARS-CoV-2 entry receptors, including ACE2 and TMPRSS2, and therefore, infection of these cells may not be the cause of the loss of the sense of smell associated with SARS-CoV-2 infection [51,53,54,64]. However, a few studies have proposed a direct infection of the OSNs by SARS-CoV-2 [65,79]. On the other hand, the infectibility of OSNs was extremely low, and it is highly unlikely that such a low level of infection could be responsible for anosmia. Importantly, no study to date has shown replication of SARS-CoV-2 in OSNs of COVID-19 patients. More recent studies of human autopsies retrieved from alive and deceased SARS-CoV-2-infected donors confirm the lack of presence of the SARS-CoV-2 Spike protein in OSNs [41,50,74]. If the OSNs are not directly infected with SARS-CoV-2 infection, then what is the cause of the temporary loss of the sense of smell in COVID-19 patients?

Given that SUS cells are a critical structural feature of the OE, loss of these cells may interfere directly with the sense of smell by affecting the uniformity of the OE or may potentially cause indirect effects on OSNs, leading to their metabolic and functional disturbance. Evidence to date suggests that loss of SUS cells caused by the viral infection leads to loss or disruption in functional and metabolic activity of supporting cells in human, mouse, and Syrian hamsters [41,50,66,74] as early as day 2 postinfection. Bryche et al. used Syrian hamsters to show desquamation of the OE, recruitment of immune cells, and loss of OSN cilia caused by the loss of SUS cells post-SARS-CoV-2 infection [50]. In another study carried out on Syrian hamsters, Zazhytska et al. showed significant damage to the OE after infection but without significant infection of the OSNs. The study also reported significant downregulation of SUS-specific markers, followed by the downregulation of OSN-specific genes and related signaling pathways that are involved in the sense of smell [74]. These observations were consistent with a study that reported the first downregulation of OR genes in the OSNs using a mouse model infected with SARS-CoV-2 [66]. Therefore, it is postulated that a persistent noncell autonomous disruption of the transcriptome occurs in the OSN following infection in the OM. Downregulation of the SUS cell-specific genes has been shown in the OE autopsies of individuals who died from COVID-19 infection [41]. Contrary to Zazhytska et al. and Ye et al., this study did not find significant downregulation of the OSN, OR genes, or the genes that are involved in odor perception. However, the study suggested that loss of support to the OSN and chemokine response are the key sequences that follow SUS cell infection. Other studies have also reported the role of immune cells in the mechanism of anosmia. SUS cell infection in the OE results in the recruitment of proinflammatory cytokines as an antiviral response. These proinflammatory cytokines may lead to OE damage, which results in dysfunction of the OSNs [50,79]. Table 2. summarizes the reported effects on olfactory sensory neurons, and Table 3. provides a summary of studies showing damage to the OE.

**Table 2:** Damage to the olfactory sensory neurons.

12 hpi	1 dpi	2 dpi	3 dpi	4 dpi	5 dpi	7 dpi	10 dpi	14 dpi	21 dpi	Olfactory Sensory Neurons Downregulation and Decrease (KE Upstream)	Ref.
☐	☒	☒	☐	☒	☐	☐	☐	☐	☐	Delayed downregulation of OSN-specific markers and their precursors is observed by 4 dpi	[74]
☐	☐	☐	☐	☒	☐	☐	☐	☐	☐	SARS-CoV-2 nuclear protein colocalizes within olfactory sensory neuron marker OMP is detected at 4 dpi	[80]
☐	☐	☒	☐	☐	☐	☐	☐	☐	☐	OSNs cilia are severely impaired after infection at 2 dpi.	[50]
☐	☐	☐	☐	☒	☐	☐	☐	☐	☐	Infected OMP+ mature OSNs were discovered at 4 dpi.	[65]

Dpi—day post inoculation; KE—key events.

**Table 3:** Summary of studies showing OE damage caused by SARS-CoV-2 at various time points after infection.

12 hpi	1 dpi	2 dpi	3 dpi	4 dpi	5 dpi	7 dpi	10 dpi	14 dpi	21 dpi	OE Damage (KE Downstream)	Ref.
☒	☐	☒	☐	☒	☐	☒	☐	☐	☐	SARS-CoV-2 N protein (NP) is scattered to indicate epithelial cell damage upon infection; damage occurs 12 h postinfection and onwards.	[80]
☐	☒	☒	☐	☒	☐	☐	☐	☐	☐	OE tissue damage but nonsignificant increase in apoptotic markers at 4 dpi.	[74]
☐	☐	☒	☐	☒	☐	☒	☒	☒	☐	SARS-CoV-2 strains UCN1 and UCN19 induce massive damage to the OE at 2 dpi, but it becomes partially healed at 14 dpi.	[50]
☐	☐	☒	☐	☒	☐	☐	☐	☒	☐	The OE shows loss of ciliation as early as 2 dpi.	[65]
☐	☐	☐	☒	☐	☒	☐	☒	☐	☒	Prominent nasal discharge from the OE was observed as early as 3 dpi but the nasal cavity normalized at the time points of 10 and 21 dpi	[81]

Dpi—day post inoculation; KE—key events.

#### 3.4.3. Modulating Factors

A recent non-peer-reviewed study reported that the OSN damage and infection by SARS-CoV-2 is more pronounced in younger individuals. Furthermore, the same study also reported delayed viral clearance, phagocytic dysfunction, and a decline in phagocytosis-related genes in biopsies of human OE from aged individuals [67]. 

### 3.5. Olfactory Sensory Neuron Genes Downregulation Leads to Short-Term Anosmia

#### 3.5.1. Biological Plausibility

Each cell type in the OE performs its role to sustain the anatomy and physiology of this tissue throughout its lifetime. SARS-CoV-2 infection in the OE has been suggested in the previous KEs to cause the death of non-neuronal cells and damage neurons. This consequently leads to loss of the sense of smell in COVID-19 patients. However, clinical data suggest that in most cases, loss of smell caused by COVID-19 is reversible. The recovery to normal olfaction potentially could be attributed to the presence of Progenitor cells in the OE, such as globose basal cells (GBCs) and horizontal basal cells (HBCs). These cells give rise individually to SUS cells and neurons, respectively [82,83,84].

#### 3.5.2. Empirical Evidence

Clinical studies report that the sense of smell is lost in the early days of SARS-CoV-2 infection, but it is not known exactly when the infection in the nasal mucosa occurs, except when human volunteers are inoculated in a controlled clinical study [85]. In this study, the loss of smell, on average, occurred at 8 days (range: 5–12 days after inoculation). This suggests that anosmia occurs once the SUS cell infection disrupts the functional and metabolic support of the odor perception pathway. One study has shown in the Syrian hamster OE that a significant OR downregulation occurs at 2 dpi and peaks at 4 dpi. This study also reported that all other OSN-specific markers and the most variable gene at earlier days recovered with the elimination of the virus at 10 dpi, except for the OR genes, i.e., Adcy3, Gng13, Cnga2, Rtp1, and Gfy for odor perception, which recovered only partially [74]. Bryche et al. have shown in hamsters that widespread SUS cell infection causes OE damage, including complete loss of cilia of the sensory neurons at 2 dpi [50]. This chemosensory loss may lead to anosmia without the complete death of the neurons.

The OE regenerates continuously through a process in which basal progenitor cells play a particularly important role. These cells, upon differentiation, replace the lost SUS cells and also rebalance the damage to the OSNs. However, the HBCs also express ACE2, and infection of these cells may slow functional recovery over a long time period [53,82]. Zazhytska et al. demonstrated transcriptional downregulation of GBCs peaking at 4 dpi after SARS-CoV-2 infection, indicating the persistence of anosmia until the viral elimination from the OE and restoration of normal transcriptional landscape and functional and metabolic profile of SUS cells [74]. Physiologically, the death and regeneration of SUS cells occur much faster than the death and regeneration of olfactory neurons [50,68,81]. Rapid replenishment of SUS cells is consistent with the rapid recovery of the sense of smell that is clinically observed in most COVID-19 cases [10].

Some studies also suggest major sensorineural olfactory loss as a mechanism of anosmia in COVID-19 patients [63,77,86,87], although there is a consensus that OSNs lack the expression of ACE2, the viral entry receptor. Furthermore, many studies have shown no or rare infection of the olfactory sensory neuron in in vivo and human ex vivo studies. However, if considering the plausibility of OSN death following direct infection of the SARS-CoV-2, it should be noted that the replacement requires 8 to 10 days [88,89,90] and an additional 5 days for cilia maturation [91]. Therefore, the extent of the damage in the OE is one possible explanation for variation in the recovery of anosmia patients. Tan et al. recently reported on the basis of a meta-analysis of the studies on the prevalence of anosmia that the sense of smell recovers in most cases of COVID-19-induced anosmia within 30 days, with the median recovery time of 14.9 days (95% confidence interval 12.7 to 20.3 days) [26].

## 4. Conclusions

Short-term COVID-19-related anosmia is often not associated with nasal obstruction. Especially with the wild-type virus, the incidence of anosmia has been observed in > 80% of COVID-19 patients [19] combined with none, mild, or more severe clinical COVID-19 symptoms. A unique clinical feature of the COVID-19 acute short-term anosmia is its transient course, which begins suddenly regardless of other features and improves gradually over a short period (about 2 weeks). This paper has described some likely mechanisms related to this reversible adverse outcome and the key role of SUS cells in this process. Recent review and meta-analysis have supported the spontaneous improvement of anosmia with a mean duration of 2–3 weeks [26,92].

**Figure 3:**
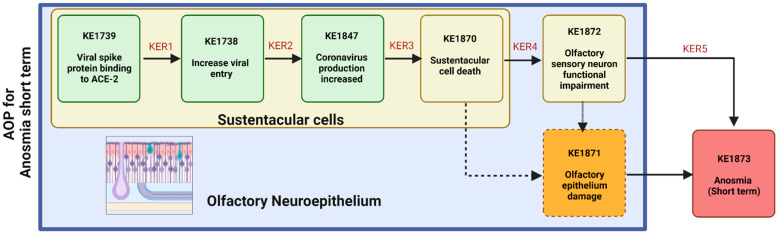
A putative AOP for anosmia induced by COVID-19 infection. KE—key event; KER—key event relationship. KE and KER refer to the AOP published at aopwiki.org/ (Accessed on 20 September 2022). Created with Biorender.com (Accessed on 20 September 2022).

In this paper, we proposed and evaluated a pathway (see Figure 3) from the binding of SARS-CoV-2 S proteins to ACE2 receptors present on SUS cells of the OE to the viral entry and production of new virions inducing a decrease in SUS cells and OSN damage leading ultimately to short-term anosmia (AOP394). Based on the evidence to date, the three following mechanisms are plausible causes for short-term anosmia in COVID-19 patients: 1) damage to the OE induced by the death of SUS cells and antiviral immune response ultimately leads to functional impairment of the olfactory system; 2) noncell autonomous downregulation of OR and OSN genes involved in odor perception; 3) chemosensory anosmia caused by the loss of sensory cilia. The late events are reversible because of the regenerative capacity of OE. However, the time of stem cell regeneration (link to the difference in regeneration time between sustentacular cells and OSNs at the level of KEs) and severity (link with viral load at the level of MIE) serve as modulating factors in complete recovery [93]. Therefore, complete recovery from short-term anosmia could take 6-8 weeks. Taking into consideration the suggested plausible causes for short-term anosmia, infection of the olfactory bulb or higher olfactory pathways is improbable.

In many patients, the process described can proceed and lead to chronic forms of anosmia characterized as long-term or permanent anosmia. Moreover, in the brain, infection of the vascular cells or the neurons implicates neuroinflammation and neurodegeneration, leading to long-term or permanent anosmia. Those cells do not have the same regeneration capacity, inducing a permanent OD. Lastly, the kinetics and dynamics of olfaction are critical feedback processes. These processes are very complex, and very particular anatomical equilibrium governs them. Considering the intricate interactions between the different cell types in the olfactory system, the specific cell type-related defense mechanisms, the immune system and the CNS, and the complexity of the relationship between CNS and the olfactory system, the interindividual differences in SARS-CoV-2 dynamic and kinetic processes are not surprising.

The AOP approach applied to long-term or permanent anosmia will be instrumental in viewing the mechanistic key event relationships to study the involvement of the higher olfactory pathways and a conductive component of olfactory disorders, as well as genetic factors and other modulating factors that may impact long-term neuro-olfactory clinical features of the COVID-19 disease. The need for continuing with AOP-based olfaction studies with wild-type and the specific mutated SARS-CoV-2 phenotypes remains urgent, especially in light of increasing numbers of olfaction-related patient presentations in hospitals around the world [94,95].

## Data Availability

Not applicable.
